# Characteristics of Particulate Matter and Volatile Organic Compound Emissions from the Combustion of Waste Vinyl

**DOI:** 10.3390/ijerph15071390

**Published:** 2018-07-02

**Authors:** Mona Loraine M. Barabad, Wonseok Jung, Michael E. Versoza, Yong-il Lee, Kyomin Choi, Duckshin Park

**Affiliations:** 1 Korea Railroad Research Institute, Uiwang City 437-757, Korea; mlmbarabad@gmail.com (M.L.M.B.); worship611@krri.re.kr (W.J.); mikeverz23@krri.re.kr (M.E.V.); freego83@krri.re.kr (Y.-i.L.); kmchoi@krri.re.kr (K.C.); 2 Railway System Engineering, University of Science and Technology, Daejeon City 34113, Korea

**Keywords:** combustion, emission, PM, VOCs, waste vinyl

## Abstract

Vinyl samples were burned in a controlled environment to determine the characteristics of particulate matter (PM) and volatile organic compound (VOC) emissions during the combustion process. Open burning of plastic or vinyl products poses several environmental and health risks in developed and developing countries, due to the release of high concentrations of harmful pollutants. The production of fine and ultrafine particles was significant. At a heat flux of 25 kW/m^2^, the production of PM of 0.35 μm in size was highest at 63.0 μg/m^3^. In comparison, at fluxes of 35 and 50 kW/m^2^, the production of PM of 0.45 μm in size was highest with values of 67.8 and 87.7 μg/m^3^, respectively. Benzene, acetone, and other toxic compounds were also identified in the analyses.

## 1. Introduction

According to the World Bank and Organization for Economic Cooperation and Development (OECD), countries generate 572 million tons of solid waste annually at an average of 2.2 kg/person/day (range 1.1–3.7) [1]. Projections for each country have been made based on the expected gross domestic product (GDP), and generation of average municipal solid waste (MSW), which are related to income level (IEA Annual Energy Outlook, 2005). Due to the increase in plastic utilization, open garbage burning occurs in areas where collection and organized waste handling measures are not implemented or are inadequate, even in urban areas, including backyard burning and open dump burning in developing countries [2,3]. In developed countries, open garbage burning is usually done in the backyards of houses in rural areas [4]. The World Bank reported that MSW has been used as fuel. Plastic, which is one waste component, comprised 0.9–9.5% of the waste in China in 1993 and 4.9% in Manila in 1997 [5]. China is a region of great concern for open burning, as it is estimated to have among the highest levels of total waste production and emissions [6]. In Tanzania, large amounts of plastic waste are commonly dumped in landfills and about 60% of the domestic solid waste is subjected to open burning daily [7]. According to the United States Environmental Protection Agency (USEPA), greenhouse gases (GHG) such as carbon dioxide (82% of all GHG emissions in the United States in 2015) are released from the incineration of municipal waste. Garbage burning is also a major source of particulate chloride and particulate matter smaller than 2.5 μm in size (PM_2.5_) in Mexico City [2]. Furthermore, it is the main global source of dioxins and several other air pollutants [8]. 

Burning plastics release high concentrations of extractable organic compounds that cause various problems in developing and developed countries. Open waste burning poses environmental and health risks to those who are exposed to the smoke. In India, landfills are notable threats, as large amounts of plastic were burned and dumped with other wastes, contributing to GHG emissions [9]. Human Rights Watch (HRW) in Lebanon reported several cases of people suffering from respiratory issues including chronic obstructive pulmonary disease (COPD), coughing, throat irritation and asthma due to inhalation of smoke from the open burning of waste [10]. It has been reported that those living near incinerators have a higher prevalence of respiratory syndromes [11], such as asthma in children in Sydney, Australia [12], and chronic exposure to waste incinerators increases cancer of the larynx [13]. The USEPA also reported that the release of particulate matter (PM) through trash burning can cause deterioration in individuals with pre-existing conditions such as bronchitis, asthma, and emphysema [14]. In addition, backyard burning releases pollutants at ground level. Thus, dilution by dispersion is minimal, which may result in accumulation of pollution near the source and high measured concentrations of pollutants (e.g., dioxins) [15,16]. A detailed summary describing the health effects of waste incineration has been published and stated that ambient concentrations of various pollutants (from waste burning) could pose health risks [17].

Particulate matter less than 10 μm diameter (PM_10_) is produced by the burning of waste and vinyl materials. This study determined the characteristics of PM_10_ and ultrafine particles produced during the combustion of vinyl in a controlled environment (cone calorimeter). Harmful compounds were also assessed, including the different volatile organic compounds (VOCs) emitted in the process.

## 2. Materials and Methods 

**Figure 1:**
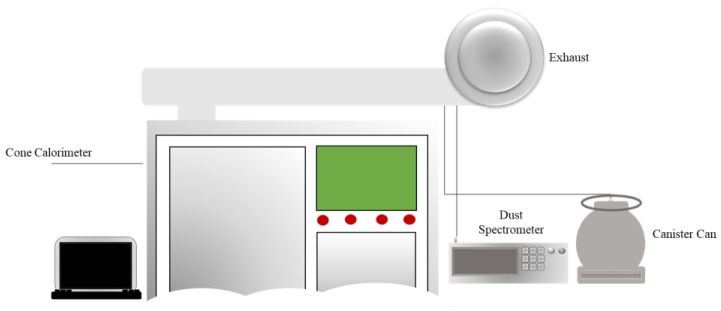
Schematic diagram showing the experimental set-up of the dual-cone calorimeter.

Figure 1 shows a schematic diagram of the experimental set-up. A dual-cone calorimeter was used to examine the production of PM and VOCs from vinyl samples burned at different temperatures. The specifics of the burning process using the cone calorimeter were given in our previous study [18]. The cone-shaped radiating electric heater can emit up to 100 kW/m^2^. In this study, the strength of the electric heater was set to 25, 35, and 50 kW/m^2^; the radiation strength of the heater was based on ISO-5660-1 as a test of the reaction to fire (heat release, smoke production, and mass loss rate). Part 1 specifically describes the HRR (heat release rate; cone calorimeter method) and smoke production rate (dynamic measurement). The mass remaining, as well as O_2_, CO and CO_2_ produced at different temperatures, were measured.

The plastic sample was collected in a rural village near Andong, a small city in South Korea. Low-density polyethylene (LDPE) plastic film is used for farming in villages. This type of waste is categorized as agricultural waste, which is collected and processed either through landfilling, recycling or incineration. Depending on the burning conditions and parameters set, a series of 2–3 burn cycles were carried out at each temperature. Vinyl samples weighing approximately 10 g were cut and positioned inside the sample holder, which measured 100 × 100 × 36 mm^3^ (W × L × H), balanced and then a final weighed was recorded. During the test, aluminum foil was placed around the holder and gas analyzers (N_2_, CO, CO_2_, and O_2_) before calibration. To control the initial conditions, the gas flow rate and concentration were also monitored. The flow rate, the weight of burned vinyl, and other parameters were stored in the software of the cone calorimeter. To measure the size contribution, an aerosol spectrometer (GRIMM 1.129 SKY OPC) with a measurement range of 0.25 to 32 μm, and regulated flow rate (6 L/min) with an external vacuum pump that was controlled by a critical orifice was used. A 6 L Restek SilcoCan stainless steel canister coated with fused silica on the inside, with its inlet aligned with the aerosol spectrometer, was used to collect compounds from the air samples. The collected pre-concentrated samples were analyzed using gas chromatography/mass spectrometry (Agilent/HP-6890; Agilent Technologies, Inc. Savage, MD, USA). 

## 3. Results

Table 1 shows the different characteristics of vinyl combustion using the cone calorimeter. The average mass of the samples was 13.0, 12.7, and 12.9 g at heat fluxes of 25, 35, and 50 kW/m^2^, respectively. 

**Figure 2:**
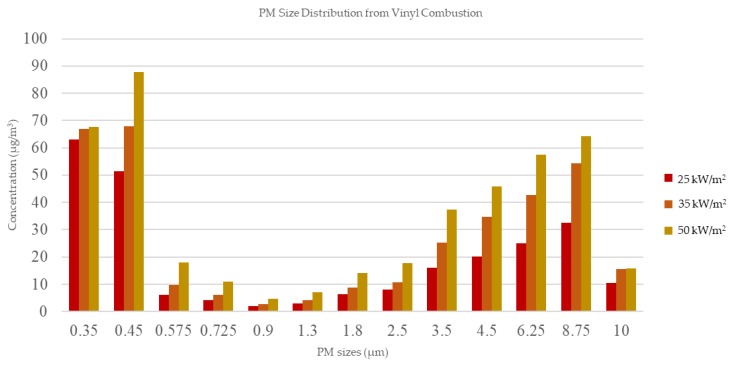
Particulate matter (PM) size distribution from vinyl combustion.

Post-combustion measurements revealed that approximately 55.9%, 59.3%, and 64.8% of the mass was removed at 25, 35, and 50 kW/m^2^, respectively. The samples produced different amounts of O_2_ consumption and percentages of CO and CO_2_. CO production was highest at 25 kW/m^2^ (31%), followed by 50 and 35 kW/m^2^ (19.3% and 7.3% CO, respectively). CO_2_ production was highest at 35 kW/m^2^ (691.8%), followed by 50 and 25 kW/m^2^ (468.5 and 462.8%, respectively). O_2_ consumption was highest at 35 kW/m^2^ (39.6 g), followed by 25 and 50 kW/m^2^ (30 g and 29.5 g respectively). O_2_ and CO_2_ values were proportional, differing from those of CO; the highest amount of CO was produced at 25 kW/m^2^, and the lowest amount was produced at 35 kW/m^2^, whereas the opposite was true for O_2_ and CO_2_. Increased oxygen consumption could lead to more combustion, which increases the release of CO_2_. However, when the O_2_ consumption rate is lower, incomplete combustion can take place, leading to soot formation. During this process, soot (C) and CO_2_ react and generate a greater percentage of CO. The maximum HRR was 330.4 ± 51.7 at 50 kW/m^2^, followed by 295.3 ± 27.6 at 35 kW/m^2^ and 247.9 ± 42.0 at 25 kW/m^2^. Figure 2 shows the PM size distribution and concentrations from vinyl combustion. The majority of particles had smaller sizes (<PM_1_) compared to PM_2.5_ to PM_10_. At 25 kW/m^2^, the peak PM size was 0.35 μm (at 63.0 μg/m^3^), whereas, the peak size at 35 and 50 kW/m^2^ was 0.45 μm, with 67.8 and 87.7 μg/m^3^, respectively. In addition, 0.45 μm had the highest peak concentration among all of the compounds, while 50 kW/m^2^ obtained the highest concentrations compared to the other heat fluxes. However, there was an apparent increase in distribution from PM_2.5_ to PM**_8.75_**, as shown Figure 2. 

The emissions factor (EF) was calculated using the following equation [15]:

(1) $$F\;\left(\frac{\mathrm{mg}}{\mathrm{kg}}\right)=\frac{\mathrm{concentration}\;\mathrm{of}\;\mathrm{pollutants}\;\left(\frac{\mathrm{mg}}{m^{3}}\right)\;X\;\mathrm{flowrate}\;\left(\frac{m^{3}}{\min\;}\right)\;X\;\mathrm{sampling}\;\mathrm{time}\;\left(\min\;\right)}{weight\;of\;burned\;vinyl\;sample\;\left(\mathrm{kg}\right)}$$

The highest EF (mg/kg) of PM in this study was for the smaller size range of 0.35–0.45 μm, as shown in Table 2. 

At 25 kW/m^2^, 0.35 μm particles had an EF of 50.4 ± 5.2 mg/kg, whereas, at 35 and 50 kW/m^2^, 0.45 μm particles had the highest EF, at 54.3 ± 1.5 and 70.2 ± 1.2 mg/kg, respectively. At a heat flux of 50 kW/m^2^, the majority of PM emissions from the vinyl combustion analysis ranged from less than PM_1_ to PM_10_.

**Figure 3:**
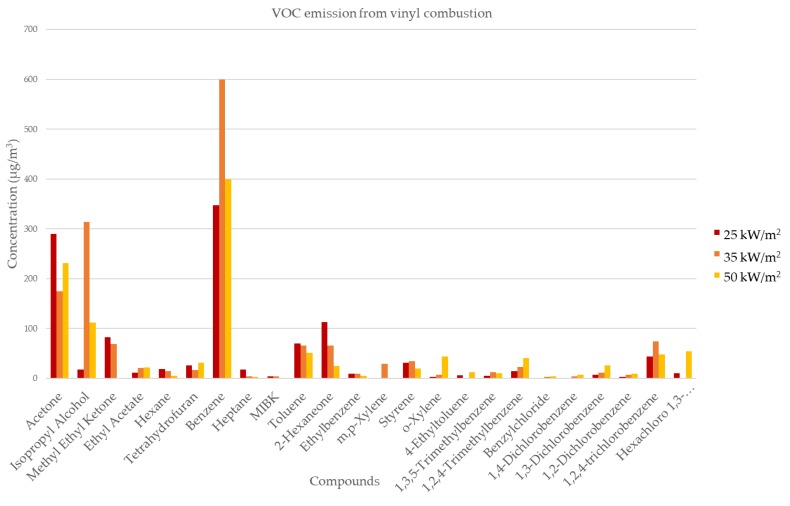
Volatile organic compound emissions from vinyl combustion.

Figure 3 shows the concentrations of VOCs from vinyl combustion at different temperatures. Of the compounds detected, benzene was the most common, comprising 30.7%, 38.3%, and 34.4% at 25, 35, and 50 kW/m^2^, respectively. Acetone comprised 26.0%, 11.2%, and 20.0% at 25, 35, and 50 kW/m^2^. Isopropyl alcohol was only produced with heat fluxes of 35 and 50 kW/m^2^, comprising 20.0% and 9.6%, respectively. By contrast, methyl ethyl ketone comprised 7.3% and 4.4% of the total at 25 and 35 kW/m^2^, respectively. 2-Hexaneone comprised 10.0%, 4.2%, and 2.3% for 25, 35, and 50 kW/m^2^, respectively, whereas toluene comprised 6.2%, 4.2%, and 4.4%. Table 3 shows the VOCs emitted in vinyl combustion and the results also varied with the heat fluxes. 

The concentrations of a few of the compounds produced exceeded 100 mg/kg. Benzene comprised 277.8 ± 14.6, 479.8 ± 19.4, and 320.0 ± 22.5 mg/kg at fluxes of 25, 35, and 50 kW/m^2^, respectively; followed by acetone at 232.0 ± 34.2, 140.0 ± 16.6, and 184.5 ± 35.0 mg/kg. The production of isopropyl alcohol also exceeded 100 mg/kg, but only at a flux of 35 kW/m^2^ with 250.8 ± 24.0. Several compounds exceeded 50 mg/kg at selected fluxes, including isopropyl alcohol at 50 kW/m^2^, methyl ethyl ketone at 25 and 35 kW/m^2^, 2-hexaneone at 25 and 35 kW/m^2^, and toluene at all three heat fluxes. Some other compounds were produced in lesser amounts or were not detected as shown in Table 4.

## 4. Discussion

Burning of waste is a source of air pollutants [19] that potentially contribute to environmental and health problems. Emission characteristics associated with the combustion of vinyl differ according to various parameters. In the current study, PM and VOC emissions from the cone calorimeter varied by combustion temperature. CO levels were lower compared to a study conducted in Mexico City, in which different plastic materials (e.g., bottles, bags, buckets) collected from several urban areas were burned; high emissions of CO were detected, which likely resulted from the burning of a high proportion of ethylene-based plastic polymers [20]. According to Font et al., CO emissions are affected by temperature; thus, when the temperature increases, carbon oxides decrease [21]. CO values were highest when a heat flux of 25 kW/m^2^ was applied. In addition, according to Lindholm et al., CO is a result of incomplete combustion and can be a major product if flame retardants disrupted the burning process [22].

In this study, PM emission characteristics were measured in a controlled environment. Of the analyzed particles, ultrafine particles (<PM_1_) occurred at the highest concentration. The total percentages (%) emitted from particles ranging in size from 0.35 μm to 0.9 μm were 53.3, 64.5, and 79.6, explaining the greater concentration of smaller-sized particles with heat fluxes of 25, 35 and 50 kW/m^2^, respectively. The total PM_10_ concentration produced at heat fluxes of 25, 35, and 50 kW/m^2^ was 237.6, 334.0, and 433.1 μg/m^3^, respectively. In this study, the characteristics of PM_10_ and PM_2.5_ can be compared to those in previous work that studied emissions from the combustion of plastic products. One study reported that 20.6 mg/g (0.020573 mg/kg) of PM_2.5_ was collected from a single waste incinerator [23]. This value was lower compared to our results, where we observed values of 190.1, 267.2, and 346.5 mg/kg with heat fluxes of 25, 35, and 50 kW/m^2^, respectively. However, other research estimated emission amounts of about 10.5 ± 8.8 g/kg [17] and 11.3 ± 7.5 g/kg [7], roughly 10 times higher than our values, which might be due to the larger sample area and amount of the materials used in that experiment. 

We also characterized VOC emissions and found that notable compounds were produced. The highest concentration of total VOC (TVOC) was obtained at a heat flux of 35 kW/m^2^ (1564.6 μg/m^3^). Results at 50 and 25 kW/m^2^ were similar (1161.4 and 1131.8 μg/m^3^, respectively). Benzene contributed the highest concentration to TVOC at 25, 35, and 50 kW/m^2^. Lemieux et al. summarized the test data generated by application of recyclers and non-recyclers for burning waste [24]. Emission products of benzene, acetone and styrene were all significant and higher than the values in our study. Styrene comprised only 2.7%, 2.7%, and 1.2% of the TVOC collected with heat fluxes of 25, 35 and 50 kW/m^2^, respectively. In addition, another study described 1,3,5 triphenylbenzene as a useful marker of the burning of plastic products in domestic waste and litter [25]; however, 1,3,5 triphenylbenzene was barely detected in our study.

Our research was conducted in the context of a controlled laboratory set-up. Several parameters were closely regulated; hence, the results may differ from actual burning conditions (i.e., open/barrel burning). Emissions from open burning can be several orders of magnitude higher compared to those of controlled combustion [24]. The results from this study presented that the heatflux characteristics could be a notable factor in the size distribution and concentration. The greatest concentrations found in this study were from ultrafine to fine particles. However, at the temperatures applied, we also observed an increase in concentrations of particles in the range of PM 3.5 μm to 8.75 μm, this trend was observed from all heatfluxes. PM emission and size distribution largely depends on the combustion conditions, where fast pyrolysis and high combustion temperatures may cause incomplete combustion [19]. Flame residence time [25] is another factor that can determine the emission products arising from incomplete combustion. To achieve complete combustion, gases produced must remain in the high-temperature zone of the furnace for a minimum residence time of 1–2 s [17]. The study can assume that when the plastic sample was under a combustion process, ultrafine and PM_10_ released with significant results. At temperatures between 700 to 800 °C, benzene and toluene were produced in significant amounts from the combustion of polyethylene (e.g., garbage bags, grain storage bags, and shopping bags) [26]. In contrast, some oxygenated compounds have also been identified in combustion experiments at low temperature [21]. Thus, temperature influences the characteristics of the particles and compounds emitted from the combustion process. However, here, the highest recorded concentration of benzene was emitted at a heat flux of 35 kW/m^2^, which was neither the lowest nor the highest temperature used in this experiment. Although this research applied different heat fluxes, the characteristics of the temperature were not taken into consideration. Furthermore, flame residence time and the characteristics of the vinyl used in the combustion process were also not examined in detail. 

Several ways of waste handling have been implemented in different countries. Developed countries apply methods such as recycling, incinerating, and discarding in landfills. Presently, as the Korean economy is rapidly growing, the immense amount of industrial waste produced is also increasing. Waste management in South Korea comprises several methods, of which incineration is one. Incineration is used to increase renewable energy production and to make use of non-recyclable and non-combustible waste (wet organic waste) [27,28]. Furthermore, incineration (in commercial incinerators, furnaces, etc.) plays an important role in industrial-waste energy production through heat energy recovery [28]. However, this study aimed to investigate the burning of plastic film, especially in the backyard context. Although household waste was reported to make up approximately 15% of the total amount of waste produced in South Korea, it is a significant target for renewable energy production. Overall, 17% (3.04 Mt) of household waste was incinerated, while recycling (57%) and landfilling (26%) were also utilized for waste management [28,29]. The Korean government introduced the volume-based waste fee (VBWF) system in 1995, in which local cities have a responsibility to collect, recycle, and treat solid waste from households, and small and large businesses. This method helped reduce waste generation and improved recycling in the MSW sector [30]. Another study investigated the removal efficiency of PM from furnaces. PM_10_ and PM_2.5_ were the primary particles produced, with coarse particles more effectively removed compared to fine particles [31]. However, developing countries are still practicing open burning and waste dumping due to inadequate disposal technology. Plastics are combustible and can result in hazardous emissions when burned in an uncontrolled environment [3]. During waste combustion, suspension of PM due to dispersion is decreased, which contributes to high concentrations of toxic pollutants. Poor dispersion of emissions will lead to an increase in direct inhalation [24], which impacts both the ambient environment and human health. 

## 5. Conclusions

Open burning of plastic and plastic related materials is still widely practiced in several countries because of poor waste handling. Here, we burned vinyl samples under controlled laboratory conditions using a cone calorimeter and analyzed the characteristics of the various pollutants emitted. 

We observed significant differences between the abundance of PM_10_ and PM_2.5_. The most abundant particle size was in the ultrafine range (<PM_1_). The smallest-sized particles (0.35 and 0.45 µm) constituted the greatest percentage of total PM emissions. Many journals have reported the characteristics of PM_10_ and PM_2.5_ emitted from waste or plastic burning. However, our investigation found that the concentration and emission characteristics of ultrafine particles were significant, which may be useful for future reference. Toxic by-products were also produced from the combustion process, including several VOCs, such as benzene, acetone, and isopropyl, which were dominant compared to other VOCs identified. 

We identified several limitations to our methodology; thus, our results may differ from those observed under actual burning conditions (i.e., garbage or open barrel burning). However, we showed that coarse, ultrafine/fine particles and carcinogenic compounds were emitted under laboratory conditions. Exposure to these pollutants may cause environmental and health issues. Although different methods of waste management have been introduced in various countries, a lack of knowledge of the end products emitted represents a hindrance to successful management. Therefore, raising public awareness of proper methods of garbage disposal is necessary. 

## Figures and Tables

**Table 1:** Combustion variables of vinyl measured using a cone calorimeter.

Heat Flux (kW/m^2^)	Peak HRR	Mass (g) + Foil	Remaining Mass	Mass Lost (g)	O_2_ (g)	CO (%)	CO_2_ (%)
25	247.9 ± 42.0	13.0 ± 1.2	5.8 ± 0.5	7.3 ± 1.0	30	31	462.8
35	295.3 ± 27.6	12.7 ± 0.7	5.2 ± 0.2	7.5 ± 0.7	39.6	7.3	691.8
50	330.4 ± 51.7	12.9 ± 0.5	4.5 ± 0.4	8.3 ± 0.2	29.5	19.3	468.5

**Table 2:** The concentration of particulate matter released in the combustion of vinyl.

PM (μm)	25 kW/m^2^	35 kW/m^2^	50 kW/m^2^
0.35	50.4 ± 5.2	53.5 ± 1.3	54.1 ± 1.0
0.45	41.2 ± 11.0	54.3 ± 1.5	70.2 ± 1.2
0.57	4.9 ± 1.6	7.8 ± 1.0	14.5 ± 2.0
0.725	3.3 ± 1.0	4.8 ± 0.5	9.0 ± 1.0
0.9	1.5 ± 0.5	2.1 ± 0.3	4.0 ± 0.2
1.3	2.5 ± 1.0	3.4 ± 0.5	6.0 ± 1.0
1.8	5.0 ± 1.5	7.0 ± 1.3	11.2 ± 1.3
2.5	6.4 ± 2.0	8.7 ± 1.5	14.2 ± 1.1
3.5	13.0 ± 3.0	20.3 ± 2.3	30.0 ± 3.2
4.5	16.2 ± 5.0	28.0 ± 4.2	37.0 ± 3.4
6.25	20.0 ± 8.3	34.2 ± 5.2	46.0 ± 6.2
8.75	26.0 ± 8.3	43.4 ± 11.0	51.5 ± 9.1
10	8.4 ± 3.3	12.5 ± 6.2	12.7 ± 3.0

**Table 3:** Experimental results (ug/m^3^) of VOC from vinyl combustion.

Compounds	25 kW/m^2^	35 kW/m^2^	50 kW/m^2^
Acetone	290.05	174.94	230.65
Isopropyl alcohol	17.10	313.45	111.81
Methyl ethyl ketone	82.72	68.67	ND
Ethyl acetate	11.60	21.20	21.73
Hexane	18.57	14.75	4.53
Tetrahydrofuran	26.17	16.54	31.03
Benzene	347.25	599.79	400.02
Heptane	17.60	4.11	3.21
Methyl isobutyl ketone	3.60	4.20	ND
Toluene	70.03	65.76	50.94
2-Hexaneone	112.90	66.19	24.93
Ethylbenzene	9.16	8.99	5.05
*m*,*p*-Xylene	0.882	28.74	1.32
Styrene	31.043	33.88	19.39
*o*-Xylene	2.97	7.14	43.27
4-Ethyltoluene	5.65	0.52	12.56
1,3,5-Trimethylbenzene	4.64	12.83	10.54
1,2,4-Trimethylbenzene	14.45	22.64	40.95
Benzyl chloride	ND	3.25	4.04
1,4-Dichlorobenzene	1.30	3.71	7.49
1,3-Dichlorobenzene	6.97	11.60	25.67
1,2-Dichlorobenzene	3.01	7.20	9.68
1,2,4-Trichlorobenzene	44.03	74.51	47.92
Hexachloro-1,3-butadiende	10.06	ND	54.71
Total:	1131.77	1564.62	1161.43

ND. Not Detected.

**Table 4:** Emission factors (mg/kg) of VOCs from the combustion of vinyl.

Compounds	25 kW/m^2^	35 kW/m^2^	50 kW/m^2^
Acetone	232.0 ± 34.2	140.0 ± 16.6	184.5 ± 35.0
Isopropyl Alcohol	13.4 ± 4.9	250.8 ± 2.4	89.4 ± 11.3
Methyl Ethyl Ketone	66.2 ± 14.0	54.9 ± 8.0	ND
Ethyl Acetate	9.3 ± 1.7	17.0 ± 2.8	17.4 ± 0.3
Hexane	14.9 ± 2.1	11.8 ± 1.4	3.6 ± 0.6
Tetrahydrofuran	20.9 ± 0.3	13.2 ± 2.9	24.8 ± 4.9
Benzene	277.8 ± 14.6	479.8 ± 19.4	320.0 ± 22.5
Heptane	14.1 ± 1.9	3.3 ± 0.4	2.6 ± 0.4
MIBK	2.9 ± 0.4	3.4 ± 0.4	ND
Toluene	56.0 ± 3.6	52.6 ± 3.4	40.8 ± 2.5
2-Hexaneone	90.3 ± 14.7	53.0 ± 1.2	19.9 ± 1.5
Ethylbenzene	7.3 ± 0.9	7.2 ± 0.8	4.0 ± 0.0
m,p-Xylene	0.7 ± 0.1	23.0 ± 0.8	1.1 ± 0.3
Styrene	24.8 ± 4.8	27.1 ± 5.4	15.5 ± 2.4
o-Xylene	2.4 ± 0.4	5.7 ± 0.4	34.6 ± 12.7
4-Ethyltoluene	4.5 ± 0.4	0.4 ± 0.1	10.0 ± 2.1
1,3,5-Trimethylbenzene	3.7 ± 0.6	10.3 ± 2.3	8.4 ± 2.6
1,2,4-Trimethylbenzene	11.6 ± 1.0	18.1 ± 2.1	32.8 ± 3.8
Benzylchloride	ND	2.6 ± 0.2	3.2 ± 0.4
1,4-Dichlorobenzene	1.0 ± 0.2	3.0 ± 0.7	6.0 ± 1.1
1,3-Dichlorobenzene	5.6 ± 0.5	9.3 ± 1.5	20.5 ± 2.0
1,2-Dichlorobenzene	2.4 ± 0.3	5.8 ± 1.1	7.7 ± 1.4
1,2,4-trichlorobenzene	35.2 ± 1.6	59.6 ± 2.5	38.3 ± 4.9
Hexachloro 1,3-Butadiende	8.0 ± 0.6	0.0 ± 1.4	43.8 ± 2.4

ND. Not Detected.

## References

[1] World Bank Urban Development Series–Knowledge Paper. https://siteresources.worldbank.org/INTURBANDEVELOPMENT/Resources/336387–1334852610766/What_a_Waste2012_Final.pdf.

[2] Li G., Lei W., Bei N., Molina L.T. (2012). Contribution of garbage burning to chloride and PM_2.5_ in Mexico City. Atmos. Chem. Phys..

[3] Estrellan C.R., Iino F. (2010). Toxic emissions from open burning. Chemosphere.

[4] USEPA (2006). An Inventory of Sources and Environmental Releases of Dioxin-Like Compounds in the United States for the Years 1987, 1995, and 2000.

[5] Rand T., Haukohl J., Marxen U. (2000). Municipal Solid Waste Incineration: A Decision Maker’s Guide.

[6] Wiedinmyer C., Yokelson R.J., Gullet B.K. (2014). Global emissions of trace gases, particulate matter, and hazardous air pollutants from open burning of domestic waste. Environ. Sci. Technol..

[7] Kassim S.M. (2006). Sustainability of Private Sector in Solid Waste Collection: A Case of Dar es Salaam Tanzania. https://dspace.lboro.ac.uk/2134/2336.

[8] Lemieux P.M., Lutes C.C., Abbott J.A., Aldous K.M. (2000). Emissions of polychlorinated dibenzo-*p*-dioxins and polychlorinated dibenzofurans from the open burning of household waste in barrels. Environ. Sci. Technol..

[9] Verma R., Vinoda K.S., Papireddy M., Gowda A.N.S. (2016). Toxic pollutants from plastic waste—A review. Procedia Environ. Sci..

[10] Human Rights Watch: “As if You’re Inhaling Your Death” (2017). The Health Risks of Burning Waste in Lebanon. https://www.hrw.org/sites/default/files/report_pdf/lebanon1117_web_1.pdf.

[11] Wang J.Y., Hsiue T.R., Chen H.I. (1992). Bronchial responsiveness in an area of air pollution resulting from wire reclamation. Arch. Dis. Child..

[12] Gray E.J., Peat J.K., Mellis C.M., Harrington J., Woolcock A.J. (1994). Asthma severity and morbidity in a population sample of Sydney school children: Part I–Prevalence and effect of air pollutants in coastal regions. Aust. N. Z. J. Med..

[13] Fingerhut M.A., Halperin W.E., Marlow D.A., Piacitelli L.A., Honchar P.A., Sweeney M.H., Greife A.L., Dill P.A., Steenland K., Suruda A.J. (1991). Cancer mortality in workers exposed to 2,3,7,8-tetrachlorodibenzo-p-dioxin. N. Engl. J. Med..

[14] EPA Web Archives. https://archive.epa.gov/epawaste/nonhaz/municipal/web/html/index-3.html.

[15] National Air Quality Management Programme (2016). Air Pollution Dispersion and Topographical Effects.

[16] Wevers M., De Fre R., Desmedt M. (2004). Effect of backyard burning on dioxin deposition and air concentrations. Chemosphere.

[17] (2000). Understanding Health Effects of Incineration—Waste Incineration and Public Health (NCBI Bookshelf).

[18] Park D.S., Barabad M.L., Lee G.J., Kwon S.B., Cho Y.M., Lee D.H., Cho K.C., Lee K.Y. (2013). Emission characteristics of particulate matter and volatile organic compounds in cow dung combustion. Environ. Sci. Technol..

[19] Maasikmets M., Kupri H.-L., Teinemaa E., Vainumae K., Arumae T., Roots O., Kimmel V. (2016). Emissions from burning municipal solid waste and wood in domestic heaters. Atmos. Pollut. Res..

[20] Christian T.J., Yokelson R.J., Cardenas B., Molina L.T., Engling G., Hsu S.-C. (2010). Trace gas and particle emissions from domestic and industrial biofuel use and garbage burning in central Mexico. Atmos. Chem. Phys..

[21] Font R., Aracil I., Fullana A., Conesa J.A. (2004). Semivolatile and volatile compounds in combustion of polyethylene. Chemosphere.

[22] Lindholm J., Brink A., Hupa M. Cone Calorimeter—A Tool for Measuring Heat Release Rate. http://www.ffrc.fi/FlameDays_2009/4B/LindholmPaper.pdf.

[23] Yan F., Zhu F., Wang Q., Xiong Y. (2016). Preliminary study of PM2.5 formations during the municipal solid waste incineration. Procedia Environ. Sci..

[24] Lemieux P.M. (1997). Evaluation of Emissions from the Open Burning of Household Waste in Barrels: Volume 1. Technical Report.

[25] Simoneit B.R.T., Medeiros P.M., Didyk B.M. (2005). Combustion products of plastics as indicators for refuse burning in the atmosphere. Environ. Sci. Technol..

[26] Font R., Aracil I., Fullana A., Gullon I.M., Conesa J.A. (2003). Semivolatile compounds in pyrolysis of polyethylene. J. Anal. Appl. Pyrolysis.

[27] Ministry of Environment (2008). Waste in Energy Strategic Plan.

[28] Ryu C. (2010). Potential of municipal solid waste for renewable energy production and reduction of Greenhouse gas emissions in South Korea. J. Air Waste Manag. Assoc..

[29] Ministry of Environment (2008). White Paper in Environment.

[30] (2006). Korea Environmental Policy Bulletin (Volume-Based Waste Fee System in Korea). http://eng.me.go.kr/eng/file/readDownloadFile.do;jsessionid=5iu1EdBTzFcpz6DbuVPif7nrjNBER2UT4RZANstftx2njZsqquNXadaD0ctMEKMy.meweb1vhost_servlet_engine3?fileId=92436&fileSeq=1.

[31] Shiota K., Tsujimoto Y., Takaoka M., Oshita K., Fujimori T. (2017). Emission of particulate matter from gasification and melting furnace for municipal solid waste in Japan. J. Environ. Chem. Eng..

